# A Boy Mesmerist

**Published:** 1891-08

**Authors:** 


### A BOY MESMERIST.

The *News*, of Detroit, Mich., gives an account of Willard McKay, a fourteen year old mesmerist, who has furnished much amusement for the spectators at Wonderland. He is probably the youngest mesmerist in the country giving public performances. Two years ago his parents moved to Marine City, and it was there, while playing with some school children, that he first discovered that he had a mesmeric power. The little boys and girls that played with Master Willard soon found that he could do as he pleased with them. His father took note of the child's power, and saw that he had the same influence over adults.

His performance at Wonderland is unique only in that the mesmerist is such a young person. Those whom he mesmerizes are absolutely in his power, and he makes them do all sorts of ridiculous things, as he wills. He says that the work does not usually tire him, but at times he gets hold of an obstreperous subject, who takes hold of something to resist his influence, and that this exhausts him. He holds as many as four persons under his influence at the same time, and makes them perform different feats.

His father, Willard McKay, who travels with him, delights to talk about his son, and has an exalted idea of what he can do. He is rather short, but bulky, and with a very mild manner. His hair is long and his whiskers are also long and of two lengths. The chin whiskers are allowed to grow untrammeled, but those from his chops are joined with a Psyche knot, hidden by his chin adornments. "Oh, it's a wonderful power my boy has," said Mr. McKay. "I am an old soldier, and contracted rheumatism in the army. Don't you know that he cured me of that? Yes, he did; he cheated the grave. When he first laid his two hands upon my forehead, I jumped as if I was shot, and hurled him from me. I couldn't stand the power. But he kept rubbing me and passing his hands over me until I was well. Oh, but he has great curing powers. And he is such a good boy. Every body likes him. Yes, we are going to travel all over the country. Does it tire him? Oh, no, he likes it."

